# Preparation of high-flowability ultra-early-strength grouting material and investigation of its mechanical properties in reinforcing fractured sandstone

**DOI:** 10.1371/journal.pone.0327032

**Published:** 2025-07-10

**Authors:** Yijiang Zong, Dong Zhu, Liang Yue, Xiangling Tao, Min Chen

**Affiliations:** 1 Jiangsu Vocational Institute of Architectural Technology, School of Transportation Engineering, Xuzhou, China; 2 China University of Mining and Technology, Xuzhou, Jiangsu, China; SASTRA Deemed University, INDIA

## Abstract

This study systematically investigated the effects of modified ultrafine fly ash, ultrafine silica fume, nano-SiO_2_, and polycarboxylate superplasticizer on the performance of cement-based grouting materials. Using a five-level orthogonal experimental, the optimal mix ratio was determined: 5.00 wt.% modified ultrafine fly ash, 10.00 wt.% ultrafine silica fume, 9.00 wt.% nano-SiO_2_, and 0.05 wt.% polycarboxylate superplasticizer. This formulation enabled the preparation of a cement-based grouting material with high-flowability and ultra-early-strength (HFUES) characteristics. Experimental results demonstrated that, compared to ordinary silicate grouting materials, the HFUES grouting material significantly enhanced the reinforcement of fractured sandstone. The reinforced specimens exhibited an average 67.42% increment in tensile strength, 123.17% increment in the uniaxial compressive strength, and 94.00% increment in elastic modulus compared to intact specimens. Microstructural analysis revealed that the grouting material was uniformly distributed within the sandstone matrix, forming a dense interfacial bond and generating a substantial amount of fibrous network-like hydration products. The volume fraction of the consolidated matrix was 1.33–1.47 times that of ordinary silicate grouting materials, while the fracture volume fraction was only 0.36–0.73 times. The study demonstrates that the HFUES grouting material exhibits excellent mechanical properties and interfacial bonding characteristics, significantly improving the reinforcement effectiveness of fractured sandstone.

## Introduction

High-flowability ultra-early-strength (HFUES) grouting materials are a new type of engineering material that combines excellent fluidity, rapid setting, and high early strength. They are widely used in emergency reinforcement and rapid repair of tunnels, mines roadway, subways, and other engineering projects [1–3]. With the rapid development of infrastructure construction, research and application of such materials have become an important direction in the field of geotechnical engineering [4,5].

HFUES grouting materials have attracted significant attention due to their high fluidity, ultra-early strength, controllable setting time, excellent bonding performance, and high strength [6–9]. These materials are based on ordinary Portland cement and incorporate functional additives such as nano-SiO_2_, ultrafine powders (such as silica fume and fly ash), and polycarboxylate superplasticizers. Those additive materials can significantly improve the material’s fluidity, early strength, and interfacial bonding properties [10–13]. However, optimizing the material’s performance involves the synergistic effects of multiple additives due to the performance trade-offs of different additives [14,15]. Therefore, orthogonal experimental design and range sensitivity analysis have been widely used to optimize material formulations and achieve an optimal balance of properties [16–19].

In the rock reinforcement engineering, researchers focus on viscosity, fluidity, early strength, long-term strength, and durability of grouting materials [20–22]. By optimizing formulations and incorporating reinforcing materials of grouting materials, the mechanical properties of grouted bodies can be significantly enhanced and satisfy the engineering requirements [23,24]. To explore the reinforcement mechanisms of grouting materials on rock mass structural surfaces, microscopic testing such as scanning electron microscopy (SEM) [25–27], X-ray computed tomography (CT) [28–30], and nuclear magnetic resonance (NMR) [31,32] have been employed.

In recent years, the development of low-carbon and environmentally friendly grouting materials has become an important trend. By reducing cement usage and utilizing industrial waste (e.g., fly ash, slag) as alternative materials, the environmental impact of engineering projects can be significantly reduced [33–35]. Overall, research on ordinary Portland cement-based grouting materials in the field of fractured rock reinforcement is rapidly advancing toward high performance, intelligence (The intelligentization of grouting materials refers to the technological advancement that endows traditional grouting materials with the ability to dynamically respond to environmental changes, autonomously adjust performance, and enable real-time monitoring and feedback through the integration of advanced material technologies, sensing technologies, data analytics, and adaptive control systems. This significantly enhances their construction precision, durability, and functionality.), durability, and environmental sustainability to meet the diverse needs of complex rock mass reinforcement projects [36–41].

Although existing research has achieved significant progress in the development of grouting materials and the reinforcement of rock mass engineering, studies on the grouting reinforcement effectiveness for fractured sandstone commonly encountered in deep underground engineering remain insufficient. To address this, this study innovatively developed a composite grouting material based on ordinary Portland cement. By incorporating modified ultrafine fly ash, ultrafine silica fume, and nano-SiO_2_ enhancers, the material achieves dual breakthroughs: In terms of material performance, the addition of glass microspheres significantly enhances the fluidity of the slurry, addressing the poor injectability of traditional materials. The synergistic effect of ultrafine silica fume and nano-SiO_2_ markedly improves the compressive strength of reinforced fractured limestone, while the gradation effect of multi-scale fillers substantially reduces the porosity of the reinforced body. Regarding engineering applicability, the initial setting time of the slurry can be dynamically adjusted by varying the content of enhancers, ensuring both construction operability and prevention of slurry leakage, while significantly enhancing the interfacial bonding strength between the material and fractured rock mass. These innovations endow the material with distinct advantages in deep roadway support and fractured roof reinforcement, demonstrating superior performance compared to traditional materials by simultaneously improving construction efficiency and reducing repair rates.

This study addresses the demand for grouting reinforcement of fractured rock masses by optimizing the formulation of high-flowability ultra-early-strength grouting materials through orthogonal experimental design. Using range analysis, the influence of various components on the key physical and mechanical properties of the material was systematically evaluated, leading to the development of a high-flowability ultra-early-strength grouting material based on ordinary Portland cement. Comparative experiments were conducted to assess the superior reinforcement effect of this material on fractured sandstone. CT scanning and SEM analysis were employed to elucidate the underlying mechanisms behind the enhanced performance of the HFUES grouting material compared to conventional Portland cement grouts. The research outcomes provide a scientific basis and technical support for material selection in the reinforcement of fractured surrounding rock in deep underground engineering.

### Study protocol and grout materials

#### Study protocol.

To ensure efficient injection of the grout into the fine fractures of fractured rock masses, the grouting material should meet the following key performance indicators: appropriate particle size distribution, excellent rheological properties, ultra-early strength characteristics, high stability, high permeability, high durability, and suitable setting time. However, a single grouting material often struggles to satisfy all these requirements simultaneously. Therefore, during the design of grouting materials, it is necessary to prioritize the performance characteristics based on the specific objectives and geological conditions, then select the most suitable materials accordingly. This study focuses on the fluidity and early strength of the grouting material for fractured surrounding rock in deep underground roadways. Thus, in adjusting the proportions of additives, the priority order is fluidity, initial setting time, the strength of the grouted matrix, and the setting time of the grout.

**Fig 1:**
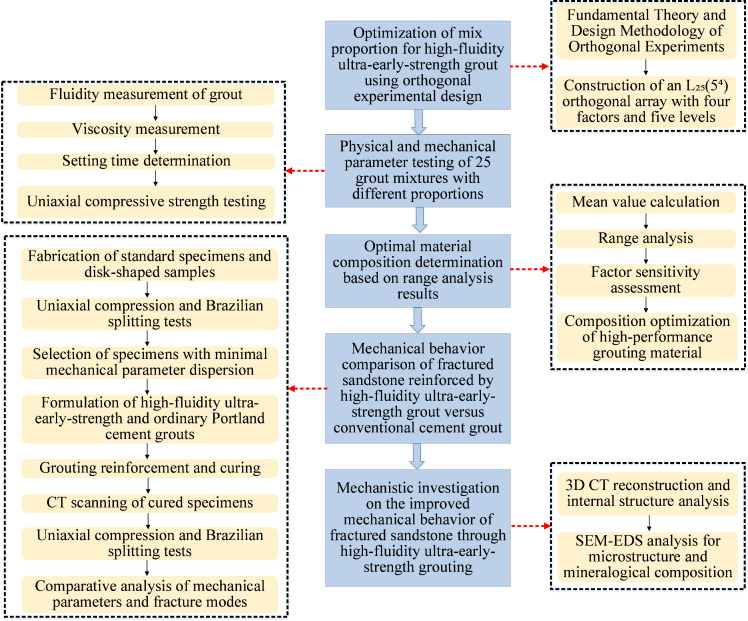
Research flow chart.

An orthogonal experimental design is used to systematically optimize the mix proportion of high-flowability ultra-early-strength grouting materials. The optimal mix ratio is first determined through orthogonal testing. Then a comparative analysis of the mechanical performance differences between the HFUES material and ordinary Portland cement material in reinforcing fractured sandstone is conducted. Finally, the underlying mechanisms for performance enhancement are thoroughly investigated. A research framework including a comprehensive technical approach encompassing material proportion optimization and performance mechanism analysis is shown in Fig 1.

### Grouting material

**Fig 2:**
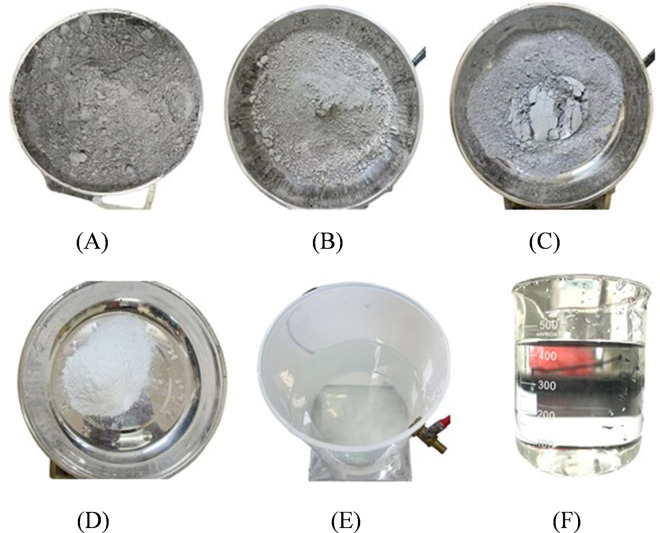
Raw materials for high-fluidity and ultra-early-strength grouting material. (A) Ordinary portland cemen; (B) Modified ultra-fine fly ash; (C) Ultra-Fine Silica Fume; (D) Nano-SiO_2_; (E) Polycarboxylate superplasticizer;(F) Purified water.

**Fig 3:**
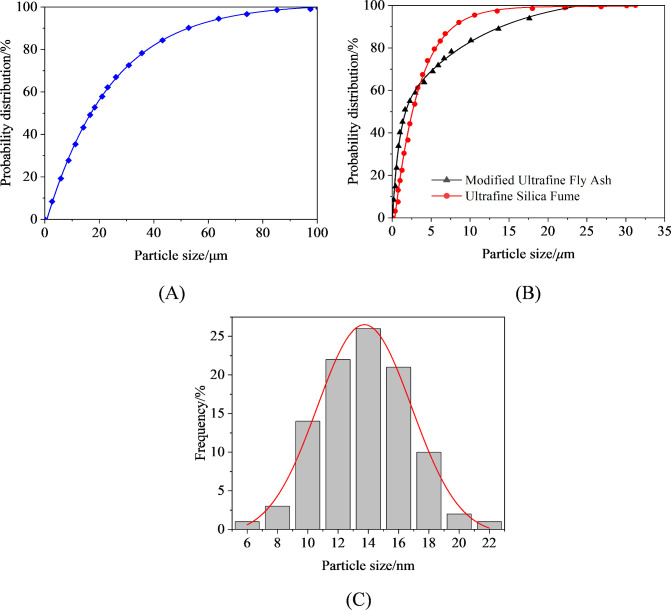
Particle size distribution curve. (A) Ordinary portland cement; (B) Modified ultra-fine fly ash and ultra-Fine Silica Fume; (C) Nano-SiO_2_.

Ordinary Portland cement (C32.5) was selected as the base material for the HFUES grouting material [42,43]. To further optimize the material’s performance, modified ultrafine fly ash, ultrafine silica fume, and nano-SiO_2_ were chosen as external enhancers, while polycarboxylate superplasticizer was used as a dispersant. The specific material composition is shown in Fig 2. The detailed chemical compositions of ordinary Portland cement, modified ultrafine fly ash, and ultrafine silica fume are provided in Table 1, the particle size distribution is shown in Fig 3.

**Table 1:** Chemical composition of components in high-fluidity and ultra-early-strength grouting material.

Components	Chemical composition (wt.%)										
	SiO_2_	Al_2_O_3_	Fe_2_O_3_	CaO	MgO	SO_3_	TiO_2_	LOSS	Na_2_O	K_2_O	C
**Ultrafine cement**	19.89	5.72	2.71	59.12	2.58	2.69	0.35	4.98	0.26	0	0
**Ultrafine fly ash**	52.32	25.28	5.96	4.58	1.62	0.71	0	0	1.49	3.52	0.85
**Ultrafine silica fume**	96.68	0.45	0.16	0.13	0.32	0.69	1.19	0	0.16	0.27	0.23
**Nano-SiO**_2_	100.00	0.00	0.00	0.00	0.00	0.00	0.00	0.00	0.00	0.00	0.00

Nano-SiO_2_ appears as a white, amorphous, flocculent, semi-transparent solid colloidal nanoparticle under normal conditions, with a particle size range of 10–20 nm and a purity of up to 99%. Due to its enormous specific surface area and nanoscale particle size, nano-SiO_2_ is prone to agglomeration in water, leading to the loss of its nano-effects. To address this issue, surface modification using the silane coupling agent KH550 is required before use, significantly enhancing its dispersibility in water.

Polycarboxylate superplasticizer is a new-generation high-efficiency dispersant, which is widely used in high-performance cementitious materials. It appears as a light-yellow transparent liquid with a density of 1.05–1.20 g/cm³ at 20°C and exhibits weak alkalinity. The chloride ion content is ≤ 0.1%, and the alkali content is ≤ 1.0%. Polycarboxylate superplasticizer demonstrates a strong dispersing effect on cement particles, significantly enhancing the fluidity and strength of cement-based materials while prolonging the retention time of workability. Furthermore, Polycarboxylate superplasticizer improves the workability and physical-mechanical properties of cementitious materials, thereby effectively enhancing engineering quality.

### Development of high-fluidity ultra-early-strength grouting material

#### Orthogonal experimental design.

To prepare high-performance high-fluidity ultra-early-strength ultrafine cement-based grouting materials, this study selected four key influencing factors for orthogonal testing: modified ultrafine fly ash (Factor A), ultrafine silica fume (Factor B), nano-SiO_2_ (Factor C), and polycarboxylate superplasticizer (Factor D). Based on relevant literature research and preliminary test results [44–47], this study determined the value ranges for each influencing factor: modified ultrafine fly ash (Factor A) ranged from 2% to 10%, ultrafine silica fume (Factor B) from 4% to 12%, nano-SiO_2_ (Factor C) from 1% to 9%, and polycarboxylate superplasticizer (Factor D) from 0.05% to 0.13%. For each factor, reasonable intervals were set and divided into five levels. A four-factor, five-level orthogonal experimental design method was adopted, with the specific test scheme presented in Table 2. During the experiments, the water-to-binder ratio was uniformly controlled at 0.6:1 to ensure consistency in testing conditions.

**Table 2:** Factor and level settings for the orthogonal experiment.

Level	Influence factor			
	Modified ultrafine fly ash (A)	Ultrafine silica fume (B)	Nano-SiO_2_ (C)	Polycarboxylate superplasticizer (D)
**1**	3%	4%	1%	0.05%
**2**	5%	6%	3%	0.07%
**3**	7%	8%	5%	0.09%
**4**	9%	10%	7%	0.11%

Orthogonal testing is a scientific experimental method based on orthogonal arrays, which efficiently analyzes the influence of multiple factors and levels through a well-designed arrangement, minimizing the number of trials. The core feature of orthogonal arrays is their orthogonality, meaning that each level of every factor appears an equal number of times in the experiments, and any combination of levels from two factors occurs with uniform frequency across all trials. This orthogonality ensures that the effects of factors can be analyzed independently, avoiding confounding, and enables rapid identification of key factors through range analysis or variance analysis.

Based on this principle, five equally spaced levels were set according to the variation range of factor values, and a four-factor, five-level orthogonal test table L25 (5⁴) was constructed, comprising a total of 25 orthogonal ratio test groups. The specific experimental design is presented in Table 3.

**Table 3:** Orthogonal experimental design L_25_ (5^4^).

Group	Modified ultrafine fly ash (wt.%)	Ultrafine silica fume (wt.%)	Nano-SiO_2_ (wt.%)	Polycarboxylate superplasticizer (wt.%)
**1**	3.00	4.00	1.00	0.05
**2**	3.00	6.00	3.00	0.07
**3**	3.00	8.00	5.00	0.09
**4**	3.00	10.00	7.00	0.11
**5**	3.00	12.00	9.00	0.13
**6**	5.00	4.00	3.00	0.09
**7**	5.00	6.00	5.00	0.11
**8**	5.00	8.00	7.00	0.13
**9**	5.00	10.00	9.00	0.05
**10**	5.00	12.00	1.00	0.07
**11**	7.00	4.00	5.00	0.13
**12**	7.00	6.00	7.00	0.05
**13**	7.00	8.00	9.00	0.07
**14**	7.00	10.00	1.00	0.09
**15**	7.00	12.00	3.00	0.11
**16**	9.00	4.00	7.00	0.07
**17**	9.00	6.00	9.00	0.09
**18**	9.00	8.00	1.00	0.11
**19**	9.00	10.00	3.00	0.13
**20**	9.00	12.00	5.00	0.05
**21**	11.00	4.00	9.00	0.11
**22**	11.00	6.00	1.00	0.13
**23**	11.00	8.00	3.00	0.05
**24**	11.00	10.00	5.00	0.07
**25**	11.00	12.00	7.00	0.09

### Measurement of physical and mechanical parameters

**Fig 4:**
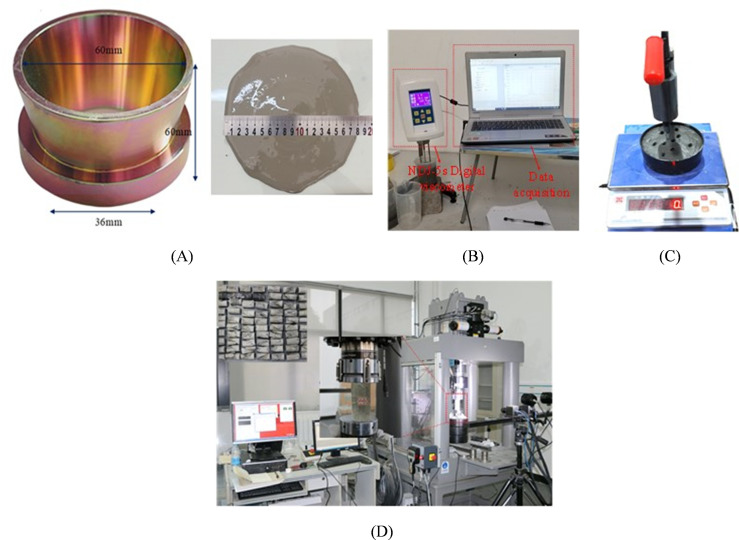
Testing of physical and mechanical parameters of grouting materials. (A) Flowability test of grouting materials; (B) Viscosity test of grouting materials; (C) Solidification test of grouting materials; (D) Mechanical properties measurement of grouting materials.

The fluidity of the grout was measured using a smooth, seamless metal truncated cone mold with dimensions of 36 mm upper diameter, 60 mm lower diameter, and 60 mm height. The well-mixed grout was slowly poured into the mold until it was completely filled, and the surface was leveled using a steel ruler. The mold was then vertically lifted, allowing the grout to flow freely on a glass plate for 30 seconds. Immediately afterward, the spread diameters of the grout in two perpendicular directions on the glass plate were measured using a ruler, as shown in Fig 4A. The average value of these two diameters was taken as the fluidity index of the grout. To minimize measurement errors, the experiments were conducted by a dedicated operator using a standardized filling method, and all tests were performed under a constant temperature of 20°C to reduce the influence of human factors and temperature fluctuations on the test results of highly fluid slurries.

In this study, the viscosity of the grouting material was measured using an NDJ-5S digital rotational viscometer with a measurement range of 1 ~ 1 × 10⁵ mPa·s. The device was equipped with a computer-based data acquisition system, enabling automatic data collection and recording through a multi-point averaging method. Prior to viscosity testing, an appropriate rotor was selected based on the estimated viscosity range of the slurry. All tests were conducted under controlled environmental conditions at a constant temperature of 20°C to minimize measurement errors. During testing, the rotational speed was initially increased gradually from 0 to 236 s^−1^ and then uniformly decreased to 0. After thorough mixing, the slurry was immediately poured into a beaker and stood for 30 s. The beaker was then adjusted to ensure the rotor was fully immersed and centered in the slurry, followed by another 30 s of equilibration before commencing data acquisition, as illustrated in Fig 4B.

The setting time of the grouting material was determined using a ZKS-100 setting time tester, as shown in Fig 4C. After standard curing for 30 minutes, the initial setting time of the grout was measured. The initial setting needle was allowed to naturally sink into the grout, and the reading was recorded after 30 seconds. Subsequently, measurements were repeated every 5 minutes, and the data were recorded. The criterion for the initial setting state was that the initial setting needle sank to a position 4 mm ± 1 mm above the bottom plate. After determining the initial setting time, the mold and grout were immediately inverted and placed in a humidity curing chamber for further curing. The initial setting needle was replaced with the final setting needle, and measurements were taken every 15 minutes. The grout was considered to reach the final setting state when the ring attachment on the final setting needle no longer left marks on the surface of the specimen, indicating that the needle had penetrated 0.5 mm into the sample. To minimize deviations in the setting time test, the consistency and surface smoothness of the grouting material were strictly controlled during the testing process.

The uniaxial compressive strength of 25 groups of hardened grout specimens cured for 2 days was measured using the MTS816 rock mechanics testing system at China University of Mining and Technology, as shown in Fig 4D. To ensure the accuracy of the test results, both end surfaces of the specimens were finely ground to achieve flatness, and a thin layer of vaseline was uniformly applied to effectively minimize the influence of end friction effects on the compressive test results, thereby reducing measurement errors introduced by human factors to the greatest extent.

### Analysis of test results

After measuring the physical and mechanical characteristic parameters of 25 groups of specimens with different mix proportions, the specific results for viscosity, initial setting time, final setting time, fluidity, and compressive strength of each group of grouting materials were summarized, as shown in Table 4. The experimental results indicate that the viscosity of the grouting materials ranged from 1680 to 4790 mPa·s, the initial setting time ranged from 85 to 176 minutes, the final setting time ranged from 168 to 278 minutes, the fluidity ranged between 23.80 and 33.80 cm, and the uniaxial compressive strength ranged from 6.04 to 8.96 MPa. Due to the wide distribution ranges of the five characteristic parameters across the 25 groups of grouting materials, the optimal mix proportion for the high-flowability ultra-early-strength ultrafine cement-based grouting material was determined using range analysis.

**Table 4:** Summary of measured characteristic parameters.

Group	Viscosity/mPa·s	Initial setting time/min	Final setting time/min	Fluidity/cm	Uniaxial compressive strength/MPa (2d)
**1**	2780	147	245	30.80	6.04
**2**	3025	162	221	29.90	7.32
**3**	3870	121	230	28.30	7.79
**4**	4360	102	190	25.50	8.05
**5**	4790	85	168	23.80	8.96
**6**	3870	176	240	30.60	7.28
**7**	3950	130	232	29.40	7.65
**8**	4250	108	198	25.90	7.98
**9**	1680	89	176	24.60	8.75
**10**	4020	115	219	29.70	7.70
**11**	3960	145	236	31.70	7.35
**12**	4160	106	218	26.50	7.85
**13**	4560	109	184	25.80	8.78
**14**	3910	127	230	30.90	7.30
**15**	4180	116	218	28.70	8.10
**16**	4020	124	224	27.80	7.75
**17**	4460	116	196	26.70	8.56
**18**	3780	125	232	32.90	6.95
**19**	4060	135	246	29.90	7.82
**20**	4270	94	196	25.60	8.08
**21**	4320	124	212	27.70	7.98
**22**	3650	135	278	33.80	6.20
**23**	3980	156	259	31.90	7.60
**24**	4160	117	227	27.20	7.84
**25**	4520	94	186	24.70	8.47

Using the range sensitivity analysis method, the mean values and ranges of performance indicators, including viscosity, initial setting time, final setting time, fluidity, and 2-day uniaxial compressive strength, were calculated and analyzed for the 25 groups of orthogonal experiments under different factors and levels.

(1) Factor sensitivity analysis on fluidity

**Fig 5:**
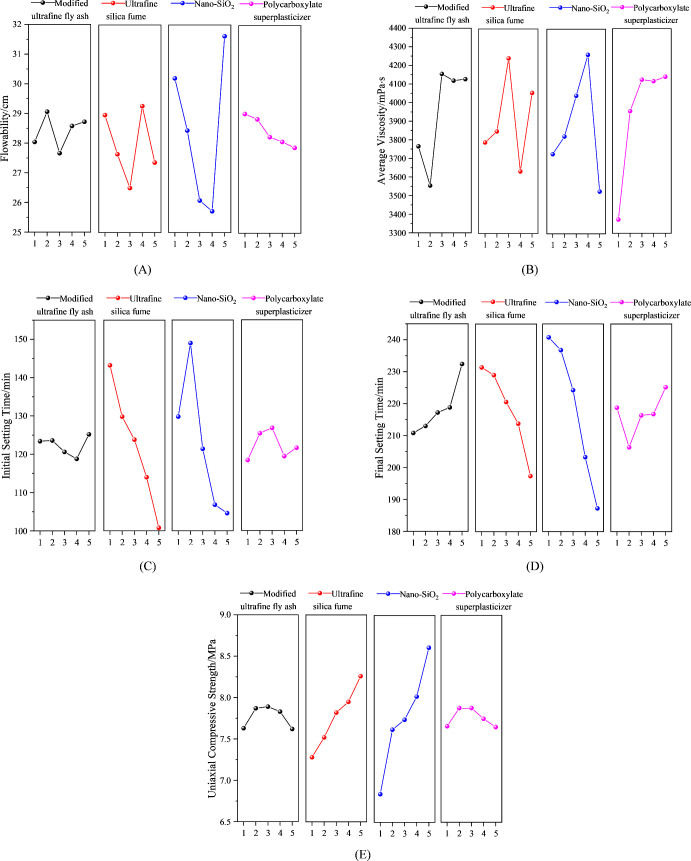
Variation trends of the influence of different factors on the physical and mechanical properties of the grout. (A) Factor levels affecting mean fluidity; (B) Factor levels affecting mean viscosity; (C) Factor levels affecting mean initial setting time; (D) Factor levels affecting mean final setting time; (E) Factor levels affecting mean 2-day uniaxial compressive strength.

From the experimental results presented in Table 5 and Fig 5A, it can be observed that the fluidity of the grouting material exhibits significant fluctuations with increasing content of modified ultra-fine fly ash and ultra-fine silica fume. This nonlinear variation is primarily attributed to the substantial differences in the content of nano-SiO_2_ and polycarboxylate superplasticizer across the five designed gradient levels. The interactions among these components result in a non-monotonic trend in fluidity.When the nano-SiO_2_ content is ≤ 7.00%, the fluidity of the grouting material demonstrates a clear negative correlation with nano-SiO_2_ content. However, when the nano-SiO_2_ content reaches 9.00%, an anomalous surge in fluidity occurs. This unexpected phenomenon may stem from two key factors: First, the high nano-SiO_2_ content intensifies particle agglomeration, significantly impairing its dispersion performance. Second, the simultaneous presence of high levels of modified ultra-fine fly ash and polycarboxylate superplasticizer at this experimental level leads to synergistic effects among multiple factors, ultimately causing the abnormal change in fluidity. Furthermore, the gradual decline in fluidity with increasing polycarboxylate superplasticizer content further corroborates the influence of multi-factor synergistic mechanisms.

**Table 5:** Range analysis of fluidity under different factor levels.

Levels	Fluidity (cm)			
	Modified ultrafine fly ash (A)	Ultrafine silica fume (B)	Nano-SiO_2_ (C)	Polycarboxylate superplasticizer (D)
**1**	28.04	28.96	30.20	**29.02**
**2**	**29.06**	27.64	28.44	28.84
**3**	27.66	26.50	26.08	28.24
**4**	28.58	**29.26**	25.72	28.08
**5**	28.72	27.36	**31.62**	27.88
**Range**	1.4	2.76	5.9	1.14

Range analysis revealed significant differences in the effects of various factors on fluidity: nano-SiO_2_ (C) and ultrafine silica fume (B) were identified as the primary influencing factors, whereas modified ultrafine fly ash (A) and polycarboxylate superplasticizer (D) exhibited relatively weaker effects. The sensitivity ranking was determined as C > B > A > D. Notably, although the difference in fluidity between level 1 and level 5 of nano-SiO_2_ was minor (Δ = 1.42 cm), the content variation reached 8 percentage points (1% vs. 9%). Based on economic analysis, the optimized ratio A2B4C1D1 was selected, as this combination ensures satisfactory fluidity performance while significantly reducing material costs.

(2) Viscosity sensitivity factor analysis

Through the analysis of data in Table 6 and Fig 5B, it can be observed that the content of modified ultrafine fly ash exhibits a distinct threshold effect on the viscosity of grouting materials: when the content is ≤ 5.00%, viscosity shows a negative correlation with the content, whereas when the content exceeds 5.00%, viscosity undergoes a sudden increase before stabilizing. This phenomenon primarily arises from two mechanisms: (1) the supercritical content significantly enhances interparticle frictional resistance, and (2) the porous structure (particularly the nano-SiO_2_ coating layer) increases the specific surface area, thereby strengthening the adsorption capacity of free water. Notably, the influence of nano-SiO_2_ and ultrafine silica fume on viscosity demonstrates a non-monotonic trend, initially increasing and then decreasing, which may be attributed to excessive addition leading to reduced particle dispersion efficiency and aggravated agglomeration. Furthermore, the impact of polycarboxylate superplasticizer is characterized by an initial rapid increase followed by stabilization, which can be ascribed to both the synergistic effect with nanomaterials and the formation of micellar structures at high concentrations, as well as delayed hydration-induced sedimentation and stratification.

**Table 6:** Range analysis of viscosity under different factor levels.

Levels	Mean viscosity (mPa·s)			
	Modified ultrafine fly ash (A)	Ultrafine silica fume (B)	Nano-SiO_2_ (C)	Polycarboxylate superplasticizer (D)
**1**	3765	3790	3728	**3374**
**2**	**3554**	3849	3823	3757
**3**	4154	4242	4042	3885
**4**	4118	**3634**	4262	3910
**5**	4126	4056	**3527**	3942
**Range**	600	608	634	568

The range analysis results indicate significant differences in the influence of various factors on viscosity: nano-SiO_2_ (C), ultra-fine silica fume (B), and modified ultra-fine fly ash (A) are the primary influencing factors, with nano-SiO_2_ and ultra-fine silica fume exhibiting particularly prominent effects. Specifically, the sensitivity ranking of the factors is as follows: C > B > A > D. Based on the above analysis, the optimal mix ratio for slurry viscosity is determined to be A2B4C5D1.

(3) Sensitivity analysis of factors affecting initial setting time

The research results (Table 7, Figs 5 C and D) indicate that the incorporation of nano-SiO_2_ and ultrafine silica fume significantly shortens the setting time of the grouting material. This phenomenon is primarily attributed to the reactive effects of these two materials: Nano-SiO_2_, owing to its enormous specific surface area, rapidly reacts with Ca(OH)_2_ to form C-S-H gel (secondary hydration reaction) while providing nucleation sites for hydration products, thereby markedly accelerating the early hydration process. Although ultrafine silica fume exhibits relatively lower reactivity, it effectively reduces the initial setting time by consuming Ca(OH)_2_. In contrast, the content of modified ultrafine fly ash and polycarboxylate superplasticizer in the grouting material has a limited influence on the initial setting time. While the micro-aggregate effect of ultrafine fly ash and the secondary reaction (pozzolanic effect) of its active components (SiO_2_/Al_2_O_3_) with cement hydration products proceed at a slower rate, the initial dilution of cement particle concentration delays the hydration process. The polycarboxylate superplasticizer, by adsorbing onto the surface of cement particles and generating electrostatic repulsion or steric hindrance, inhibits the initial hydration of cement, resulting in a retarding effect. However, the synergistic effect of nano-SiO_2_ and ultrafine silica fume alters the influence mechanism of fly ash and superplasticizer on the setting time when used individually.

**Table 7:** Mean and range of coagulation time across different factor levels.

Levels	Modified ultrafine fly ash (A)		Ultrafine silica fume (B)		Nano-SiO_2_ (C)		Polycarboxylate superplasticizer (D)	
	IST (min)	*t*_*f*_ (min)	*t*_*i*_ (min)	*t*_*f*_ (min)	*t*_*i*_ (min)	*t*_*f*_ (min)	*t*_*i*_ (min)	*t*_*f*_ (min)
**1**	123.40	**210.80**	143.2	231.4	129.80	240.80	**118.40**	218.80
**2**	123.60	213.00	129.80	229.00	149.00	236.80	125.4	**206.40**
**3**	120.60	217.20	123.80	220.60	121.40	224.28	126.80	216.40
**4**	**118.80**	218.80	114.00	213.80	106.80	203.30	119.40	216.80
**5**	125.20	232.40	**100.80**	**197.40**	**104.60**	**187.26**	121.60	225.20
**Range**	6.40	21.60	42.40	34.00	44.40	53.54	8.40	18.80

*t*_*i:* Initial setting time;_ *t*_*f:* Final setting time._

The range analysis revealed that the sensitivity of each factor to the setting time followed the order: C (nano-SiO_2_) > B (ultrafine silica fume) > D (polycarboxylate superplasticizer) > A (modified ultrafine fly ash). Based on this, the optimal mix proportion for the initial setting time of the slurry was determined as A4B5C5D1, while the optimal mix proportion for the final setting time was A1B5C5D2. These results provide important guidance for the performance optimization of grouting materials.

(4) Sensitivity analysis of factors affecting 2-day uniaxial compressive strength

From Table 8 and Fig 5E, it can be observed that the 2-day uniaxial compressive strength of the grouting material exhibits a significant positive correlation with the content of nano-SiO_2_ and ultrafine silica fume. This phenomenon is primarily attributed to the extremely small particle size and large specific surface area of nano-SiO_2_ and ultrafine silica fume, which enable them to rapidly react with Ca(OH)_2_, a hydration product of cement, to form C-S-H gel, thereby significantly promoting the development of early strength.In contrast, the influence of modified ultrafine fly ash and polycarboxylate superplasticizer content on the 2-day uniaxial compressive strength follows a similar trend: the initial strength increases slowly with increasing content, stabilizes after reaching a certain threshold, and then exhibits a gradual decline. This variation may stem from the micro-bubble effect introduced by excessive polycarboxylate superplasticizer. When the bubble content exceeds a critical value, it adversely affects the material strength.

**Table 8:** Range analysis of initial setting time under different factor levels.

Levels	Uniaxial compressive strength (MPa) (2d)			
**Modified ultrafine fly ash** (**A)**	**Ultrafine silica fume** (**B)**	**Nano-SiO**_2_ (**C)**	**Polycarboxylate superplasticizer** (**D)**
**1**	7.63	7.28	6.84	7.66
**2**	7.87	7.52	7.62	**7.88**
**3**	**7.89**	7.82	7.74	7.88
**4**	7.83	7.95	8.02	7.75
**5**	7.62	**8.26**	**8.61**	7.65
**Range**	0.27	0.98	1.77	0.23

The range analysis results indicate that the sensitivity of each factor to the 2-day uniaxial compressive strength follows the order: C (nano-SiO_2_) > B (ultrafine silica fume) > A (modified ultrafine fly ash) > D (polycarboxylate superplasticizer). Based on the range analysis results, the optimal mix proportion for maximizing the 2-day uniaxial compressive strength of the slurry is determined as A3B5C5D2.

The design of high-flowability ultra-early-strength grouting materials must prioritize meeting the requirements of high fluidity and early strength. Therefore, when optimizing the mix proportion, the viscosity, initial setting time, and 2-day compressive strength of the grout are considered as the primary factors, while the final setting time and fluidity are secondary factors. The specific analysis is as follows:

Modified ultrafine fly ash (Factor A): When fluidity is the primary factor, modified ultrafine fly ash plays a major role; however, for the other four indicators (viscosity, initial setting time, final setting time, and 2-day compressive strength), its influence is secondary. For the 2-day compressive strength indicator, the range value of A2 is 7.87 MPa, which meets the strength requirements. Therefore, considering the influence of fluidity, A2 is selected.

Ultrafine silica fume (Factor B): It is a primary factor for fluidity and 2-day compressive strength but a secondary factor for the other three indicators (viscosity, initial setting time, and final setting time). For the 2-day compressive strength indicator, the range value of B4 is 7.95 MPa, fully satisfying the strength requirements. Thus, B4 is selected.

Nano-SiO_2_ (Factor C): It is a primary factor for viscosity and 2-day uniaxial compressive strength but a secondary factor for the other three indicators (initial setting time, final setting time, and fluidity). Since C5 achieves optimal performance in viscosity, initial setting time, and fluidity, C5 is selected.

Polycarboxylate superplasticizer (Factor D): It is a primary factor for fluidity and 2-day uniaxial compressive strength but a secondary factor for the other three indicators (viscosity, initial setting time, and final setting time). The range values of D1 and D2 for the 2-day compressive strength indicator are similar. From an economic perspective, to reduce the dosage of the polycarboxylate superplasticizer, D1 is selected.

In summary, the optimal mix proportion for the HFUES grouting material is A2B4C5D1. Through comprehensive balance analysis, the optimal mix proportion is determined as follows: 5.00 wt.% modified ultrafine fly ash, 10.00 wt.% ultrafine silica fume, 9.00 wt.% nano-SiO_2_, and 0.05 wt.% polycarboxylate superplasticizer. Using a water-to-binder ratio of 0.6:1.0, the performance indicators of the optimized HFUES grouting material and ordinary Portland cement grout were tested, and the results are shown in Table 9.

**Table 9:** Performance test results of high-flowability ultra-early-strength and ordinary portland cement grouts.

Designation	Viscosity/mPa·s	Initial setting time/min	Final setting time/min	Fluidity/cm	Uniaxial compressive strength/MPa
**High-flowability ultra-early-strength grout**	3580	102.50	192.48	29.18	8.42
**Ordinary Portland cement**	4850	179.40	385.68	21.65	6.17

As shown in Table 9, the performance of the HFUES grouting material is significantly superior to that of ordinary Portland cement grout. Specifically, the decrement of viscosity, initial and final setting times are 26.12%, 42.85% and 50.09%, respectively. The increment of the fluidity and 2-day uniaxial compressive strength is 34.78% and 36.47%, respectively.

### Grouting reinforcement effects and mechanisms

#### Design of grouting reinforcement scheme.

Red sandstone was selected to obtain the fractured specimens to systematically study the performance differences between HFUES and ultrafine ordinary Portland grouting materials in fractured reinforcement effect. The main mineral components of the red sandstone include quartz, feldspar, cementing materials, iron oxides, and clay minerals, exhibiting a light red color with an average density of 2.35 × 10^3^ kg/m^3^ under natural conditions.

**Fig 6:**
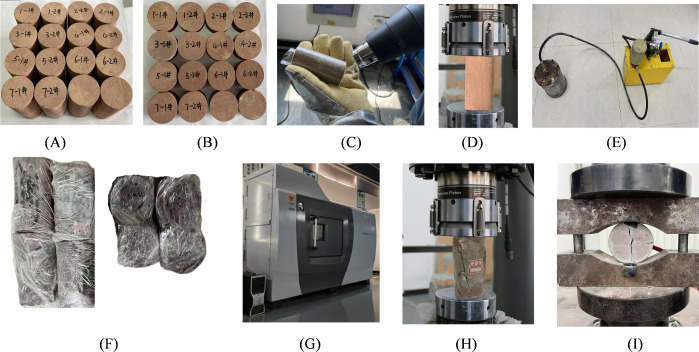
Scheme and procedure for grouting reinforcement of fractured red sandstone. (A) Standard red sandstone specimen; (B) Disk-shaped specimen; (C) Thermoplastic sealing of specimens for protection; (D) Specimen failure test; (E) Specimen grouting; (F) Natural conservation of specimens; (G) CT testing; (H) Uniaxial compression test after reinforcement; (I) Modified Brazilian splitting test after reinforcement.

The experimental study primarily focused on measuring the uniaxial compressive strength and tensile strength of the specimens. To ensure the accuracy of the result, 16 standard specimens (Fig 6A) (diameter φ = 50 ± 1 mm, height h = 100 ± 1 mm) and 16 Brazilian disc specimens (Fig 6 B) (diameter φ = 50 ± 1 mm, thickness t = 25 ± 1 mm) were prepared. Given the brittleness of the specimens are prone to sudden fracturing or spalling during the experiment, which may compromise subsequent grouting reinforcement experiments, a thermoplastic encapsulation protection treatment was applied before fracture testing, as illustrated in Fig 6 C. After completing the failure tests (Fig 6 D), all data was analyzed to select four standard specimens and four Brazilian disc specimens with the least variability for grouting reinforcement. HFUES and ultra-fine ordinary Portland cement were employed for grouting materials. The corresponding reinforced specimens were labeled as 2−1#, 2−2# and 4−1#, 4−2#, respectively.

The grouting reinforcement test was conducted using the self-developed JXT-01 manual hydraulic grouting system (Fig 6 E). This system consists of a pressure system, pressure cylinder, instrumentation, and control valves. Its grouting pressure range is 0–10 MPa with precisely controllable grouting rates, making it suitable for precise grouting operations on small-scale specimens. Based on the practical conditions of field grouting engineering, the grouting pressure was set at 2 MPa with a pressure-holding time of 30 min.

After grouting, the specimens were first subjected to 24 h of film-wrapped natural curing (Fig 6 F), followed by 24 h of standard curing. After the standard curing, CT scanning tests (Fig 6 G) were performed to observe the distribution characteristics of the two grouting materials’ solidified bodies inside the specimens. Then quantitatively analyzed the proportions of internal fractures and grout solidification. Finally, uniaxial compression (Fig 6 H) and Brazilian splitting tests (Fig 6 I) were conducted using the MTS816 electro-hydraulic servo-controlled rock mechanics testing system to comparatively analyze the reinforcement effects of the two grouting materials on fractured specimens.

### Analysis of grouting reinforcement effectiveness

**Fig 7:**
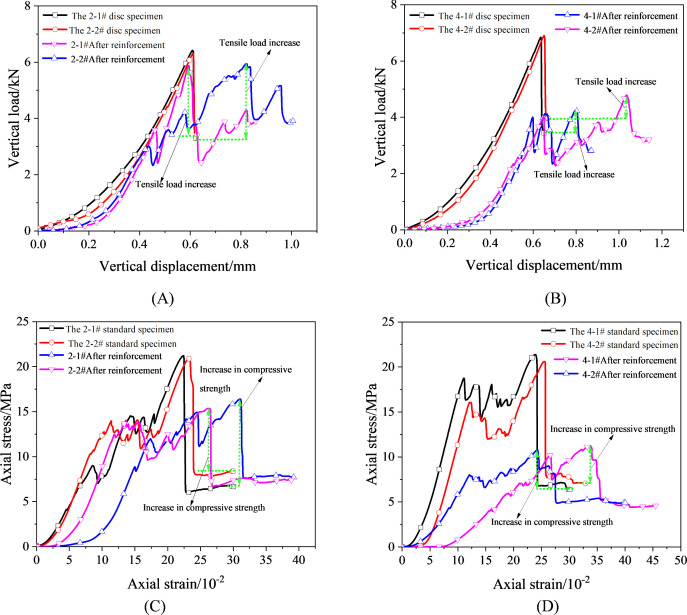
Uniaxial compression and brazilian splitting test curves of intact and grout-reinforced specimens. (A) Tensile stress curves of disc specimens before and after reinforcement with high-flowability ultra-early-strength grouting material; (B) Tensile stress curves before and after reinforcement with ordinary portland cement grouting material; (C) Uniaxial compression curves before and after reinforcement with high-flowability ultra-early-strength grouting material; (D) Uniaxial compression curves before and after reinforcement with ordinary portland cement grouting material.

Fig 7 presents the mechanical response curves of intact specimens and standard/disk specimens after fracture grouting reinforcement. The experimental results indicate that the optimized and screened specimens exhibit minimal testing variability. Taking the 2−1# and 2−2# disk specimens as examples, the tensile loads of the two intact specimens were 6.42 kN and 6.30 kN, respectively, with a coefficient of variation (COV) of only 0.94%. The maximum displacements were 0.62 mm and 0.63 mm, respectively, with a remarkably low COV of 0.08%. The residual tensile loads were 3.28 kN and 3.42 kN, respectively, with a COV of 3.30%. These data demonstrate that the optimized and screened red sandstone specimens possess excellent uniformity and reliability, indicating they were suitable for quantitative analysis of the reinforcement effects of two grouting materials on fractured red sandstone specimens. This provides a reliable experimental foundation for subsequent research.

From Figs 7 A and B, it can be observed that intact red sandstone disk specimens exhibit typical brittle characteristics during the Brazilian splitting test, with the force-displacement curve showing a vertical drop after reaching peak strength. In contrast, the fractured specimens reinforced with two types of grouting materials display noticeable stress fluctuations both before and after the peak tensile strength during the Brazilian splitting process, indicating significant plastic characteristics in the reinforced specimens. Notably, specimens reinforced with HFUES grouting material exhibit strain hardening behavior after the peak, while those reinforced with ultra-fine ordinary Portland grouting material show strain softening behavior after the peak. This different demonstrates that the tensile performance of reinforced specimens with HFUES grouting material is significantly superior to that with ordinary Portland grouting material.

In the uniaxial compression test, standard specimens exhibit obvious stress drops and fluctuations in the stress-strain curve before reaching peak strength. This phenomenon suggests that the mineral particles within the specimens are primarily bonded through cementation. When the vertical load reaches a certain level, the cementation structure gradually fails, leading to significant stress fluctuations. Consistent with the Brazilian splitting test results, fractured specimens reinforced with two grouting materials exhibit clear plastic failure characteristics. However, there are notable differences in the stress-strain curves between the two groups of specimens. Specifically, the pre-peak curves of standard specimens reinforced with HFUES grouting material is similar to those of intact specimens, while the post-peak curve shows strain hardening behavior, as shown in Fig 7 C. In contrast, specimens reinforced with ultra-fine ordinary Portland grouting material exhibit mechanical characteristics before the peak that deviate significantly from those of intact specimens, along with strain softening behavior after the peak, as shown in Fig 7 D. These results indicate that HFUES grouting material significantly outperforms ordinary Portland grouting material in terms of uniaxial compression mechanical properties, providing important experimental evidence for the selection of grouting materials in engineering practice.

**Fig 8:**
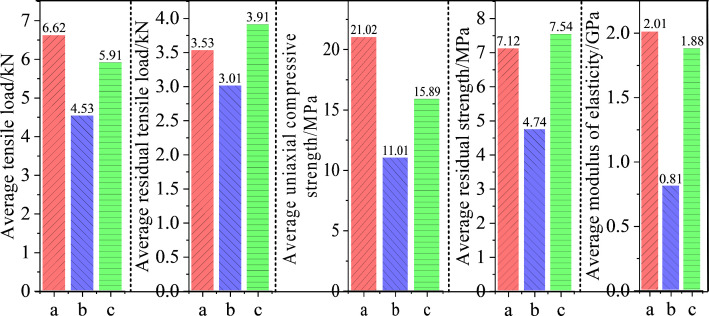
Bar charts of mechanical parameters for intact and grout-reinforced specimens. a- intact specimens; b- specimens reinforced with ordinary Portland cement grout; c- specimens reinforced with high-flowability ultra-early-strength grout.

Based on the test results in Fig 7, the mechanical parameters of the specimens were summarized in Table 10. Then bar charts were plotted to illustrate the changes in the mechanical parameters of the specimens, as shown in Fig 8, where *a* represents the average mechanical parameters of the four intact specimens. As shown in Fig 8, there is a significant difference in the average tensile strength improvement rate between the fractured specimens reinforced with the two grouting materials. Compared to ordinary Portland cement grouting material, the average tensile load of fractured red sandstone reinforced with HFUES grouting material increased by 30.46%, reaching 89.27% of the average tensile load of intact specimens. Compared to the average tensile residual load of intact specimens, the average tensile load improvement rates for specimens reinforced with HFUES and ordinary Portland cement grouting materials were 67.42% and 28.32%, respectively. Their residual tensile loads reached 110.76% and 85.27% of those of intact specimens, respectively.

**Table 10:** Statistical Table of Mechanical Parameters for Intact and Grout-Reinforced Specimens.

Number	Intact specimens					Grout-reinforced specimens				
	Tensile load (kN)	Residual tensile load (kN)	Compressive strength (MPa)	Elastic modulus (GPa)	Residual strength (MPa)	Tensile load (kN)	Residual tensile load (kN)	Compressive strength (MPa)	Elastic modulus (GPa)	Residual strength (MPa)
**2−1#**	6.42	3.28	21.21	1.92	6.67	5.87	3.90	16.40	1.98	7.69
**2−2#**	6.30	3.42	20.88	1.85	8.37	5.94	3.91	15.37	1.78	7.38
**4−1#**	6.84	3.44	21.37	2.09	6.39	4.26	2.82	11.26	1.03	4.58
**4−2#**	6.90	3.97	20.61	2.15	7.06	4.79	3.20	10.75	0.59	4.90

Fig 8 presents the results of the uniaxial compression tests. The average residual strength of intact standard specimens was 7.12 MPa, while the average compressive strengths of fractured specimens reinforced with HFUES and ordinary Portland cement grouting materials were 15.89 MPa and 11.01 MPa, respectively, corresponding to 75.59% and 52.38% of the average compressive strength of intact specimens. Compared to the average residual strength of intact specimens, the strength improvement rates of the reinforced specimens were 123.17% and 54.63%, respectively. Additionally, the residual strengths of the reinforced fractured red sandstone specimens under uniaxial compressive were 7.54 MPa and 4.74 MPa, reaching 105.90% and 66.57% of the residual strength of intact specimens, respectively. The above results mean that HFUES grouting material significantly enhanced the uniaxial compressive strength of the fractured red sandstone specimens. The enhancement effect may result from the effective filling of voids between red sandstone particles by the consolidated grout, which improved the overall compactness and load-bearing capacity of the structure.

Besides, the elastic moduli of specimens reinforced with high-flowability ultra-early-strength and ordinary Portland cement grouting materials reached 94.00% and 40.50% of the average elastic modulus of intact specimens, respectively. Notably, the elastic modulus of specimens reinforced with HFUES grouting material was the closest to that of intact specimens, demonstrating that this grouting material significantly enhances the stiffness of fractured red sandstone. The addition of the grouting material effectively improved the integrity of the fractured surfaces of the red sandstone, significantly reducing the deformation of the specimens under external forces, thereby enhancing their mechanical properties and structural stability.

**Fig 9:**
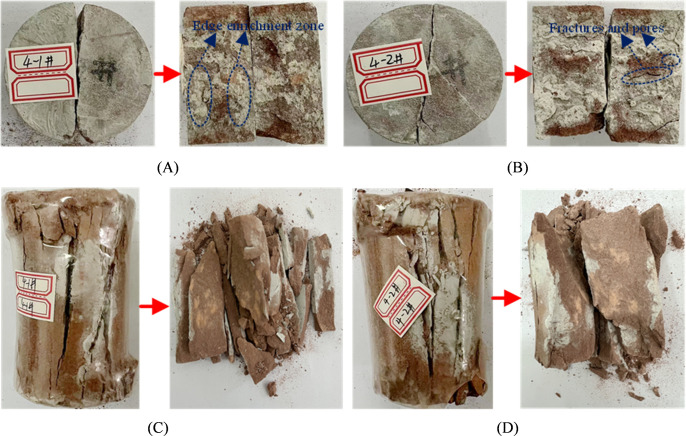
Failure modes of disk and standard specimens after reinforcement of 4#. (A) 4−1# disc specimen; (B) 4−2# disc specimen; (C) 4−1# standard specimen; (D) 4−2# standard specimen.

Fig 9 shows the final failure modes of fractured specimens reinforced with ordinary Portland cement grouting material. As shown in Figs 9 A and B, the reinforced fractured disc specimens still failed along the grout-rock interface during the Brazilian splitting test. The failure surface reveal that the consolidated body of ordinary Portland cement grouting material is unevenly distributed, with obvious localized enrichment at the edges of the fracture surface. The consolidated grout primarily adheres to the surfaces of rigid sandstone particles with high roughness, while the central region of the fracture surface exhibits sparse and limited grout consolidation. Additionally, the interface between the ordinary Portland cement consolidated body and the sandstone matrix is distinct, with a noticeable transition zone and numerous microcracks distributed at the interface.

From Figs 9 C and D, it can be observed that the fractured red sandstone standard specimens reinforced with ordinary Portland cement grouting material mainly exhibit splitting failure under uniaxial compression. The cracks primarily propagate along the original fracture surface, eventually dividing the specimen into blocks and flakes. Due to the poor fluidity of ordinary Portland cement grouting material, the grout failed to fully fill the fracture surface. Observations of the specimen unfolding in Figs 9 C and D show that the consolidated grout only covers the edge regions of the fracture surface, with limited coverage in the internal regions, indicating that most of the fracture surface was not effectively filled by the ordinary Portland cement grout.

**Fig 10:**
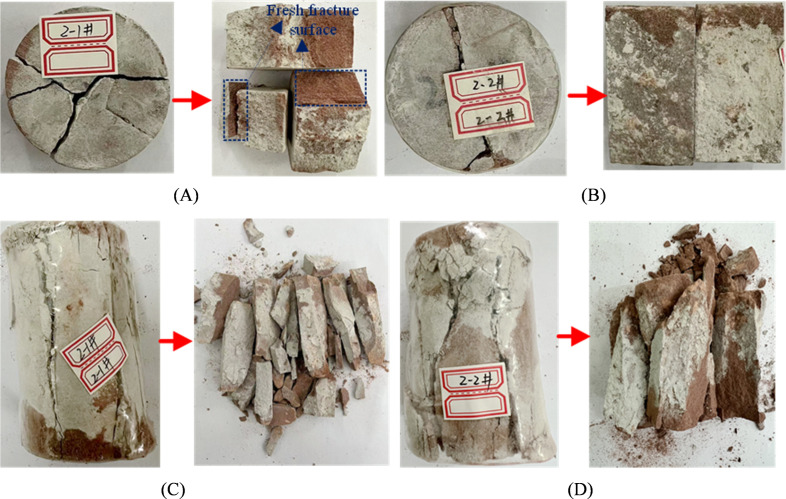
Failure modes of disk and standard specimens after reinforcement of 2#. (A) 2−1# disc specimen; (B) 2−1# disc specimen; (C) 2−1# standard specimen; (D) 2−2# standard specimen.

As shown in Figs 10 A and B, compared to the reinforcement effect of ordinary Portland cement grouting material, the consolidated body of HFUES grouting material is more uniformly distributed at the interface, with significantly reduced localized enrichment. This phenomenon is closely related to the improved fluidity and permeability provided by the nanomaterial, enabling the grout to effectively fill the fracture surfaces, internal cracks, and voids of the sandstone. The interface between the consolidated body and the sandstone matrix shows no distinct transition zone, and the consolidated body exhibits a dense structure with strong bonding to the rock surface. That may result from the fine hydration products in the HFUES grouting material. After Brazilian splitting test, the specimens not only failed along the original fracture surface but also generated new fracture surfaces (Fig 10 A), indicating the HFUES grouting material significantly improved the tensile strength of the fractured sandstone. The tensile capacity of the grout-rock interface approached that of intact specimens.

The failure modes of fractured standard specimens reinforced with HFUES grouting material in uniaxial compression exhibiting stronger ductile characteristics and more complex failure patterns than those reinforced with ordinary Portland cement. The failure modes of the specimens are dominated by shear failure, with cracks typically propagating diagonally, ultimately forming bulging failure bodies (as shown in Figs 10 C and D). The unfolding results shown that the failure surfaces are covered with a wide range of tightly bonded consolidated grout, indicating high interfacial bonding strength. Compared to specimens reinforced with ordinary Portland cement, those reinforced with HFUES grouting material exhibit more uniform failure modes, demonstrating better overall stability and resistance to failure.

### Discussion on the mechanism of grouting reinforcement effects

**Fig 11:**
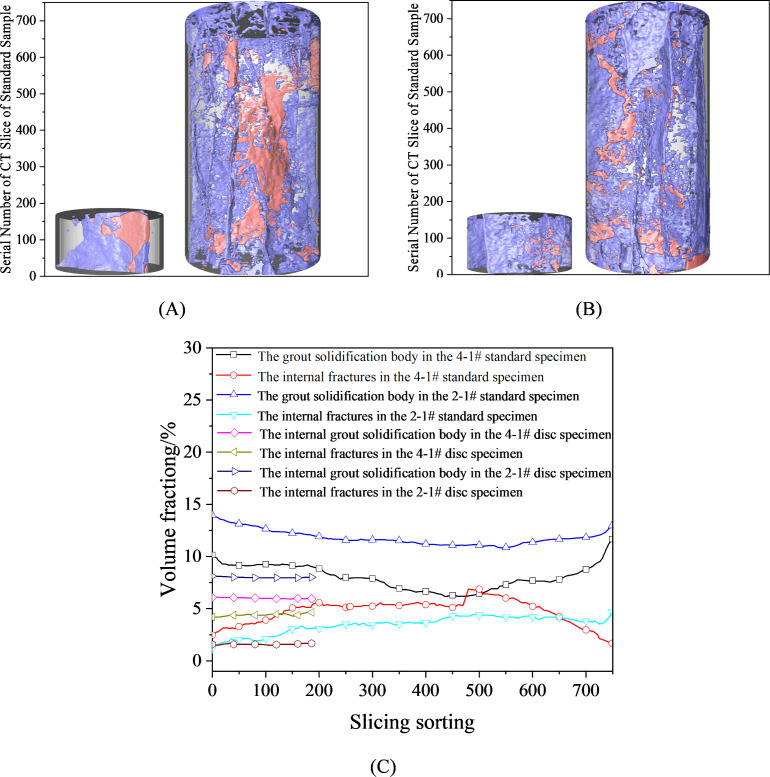
The 3D reconstruction structure diagrams, fracture networks, and volume fractions of calculi in specimens under two treatment schemes. (A) Three-dimensional reconstruction image of 4−1# specimen after fracture reinforcement; (B) Three-dimensional reconstruction image of 2−1# specimen after fracture reinforcement; (C) Volume fractions of internal fractures and grout consolidation in red sandstone specimens after grouting reinforcement.

To further explore the differences in mechanical properties between reinforced fractured specimen with two grouting materials, this study conducted a detailed analysis of the specimens using computed tomography (CT) scanning technology [48–51]. A total of 187 and 750 slices were cut perpendicular to the axial direction of the Brazilian disc and standard specimens, respectively, with each slice having a thickness of approximately 0.21 mm. The slices were numbered sequentially from bottom to top as 1 → 187# and 1 → 750#. Three-dimensional reconstruction and quantitative analysis were performed based on the slice sequences, as shown in Fig 11.

Fig 11A and B show the three-dimensional reconstruction images of fractured red sandstone specimens reinforced with different two grouting materials, respectively. In these images, the red regions represent fracture distributions, and the blue regions represent grout consolidation distributions. The CT images indicate that, the HFUES grouting material can more effectively fill the fractured surfaces of red sandstone, forming a continuous and dense consolidated structureand finally reducing the aperture and connectivity of internal fractures. Additionally, the consolidated body in the 2−1# specimen is more uniformly, continuously, and densely distributed. That can significantly enhance the integrity and stability of the fractured red sandstone specimen, enabling it to better withstand external loads.

Using threshold segmentation technology, the volume fractions of pores and grout consolidation within each slice of the specimens were accurately extracted, and the results were statistically analyzed according to the slice numbering sequence, as shown in Fig 11C. The volume fractions of the consolidated body and fractures within the Brazilian disc specimens show small variations along the axial direction, indicating a relatively uniform distribution. This uniformity is primarily attributed to the simple fracture pattern and single fracture surface of the specimens, allowing the grouting material to diffuse evenly. Specific data show that the average volume fraction of the consolidated body and fracture in the 2−1# specimen are 7.99% and 1.58%, respectively. While the corresponding values for the 4−1# specimen are 6.01% and 4.38%, respectively. Comparative analysis reveals that the consolidated body volume fraction of the 2−1# specimen is 1.33 times that of the 4−1# specimen, while the fracture volume fraction is only 0.36 times that of the 4−1# specimen. These results are highly consistent with the tensile strength characteristics and the macroscopic distribution of the consolidated body on the fracture surfaces, further validating the intrinsic relationship between grouting effectiveness and the tensile performance of the specimens.

The red sandstone standard specimens exhibit more complex fracture patterns during uniaxial compression, with diverse fracture surfaces, making it difficult for the grout to diffuse uniformly. As shown in Fig 11C, the volume fraction of the consolidated body in the edge regions of the standard specimens is significantly higher than that in the internal regions, while the fracture volume fraction shows the opposite trend. The average volume fraction of the consolidated body and fracture in the 2−1# standard specimen are 11.79% and 3.48%, respectively. While the corresponding values for the 4−1# specimen are 8.00% and 4.79%, respectively. Comparative analysis indicates that the consolidated body volume fraction of the 2−1# specimen is 1.47 times that of the 4−1# specimen, while the fracture volume fraction is only 0.73 times. These results are highly consistent with the compressive strength characteristics of the reinforced specimens, further demonstrating that the HFUES grouting material has a significant reinforcement effect on fractured sandstone specimens, with performance superior to that of ordinary Portland cement grouting material.

**Fig 12:**
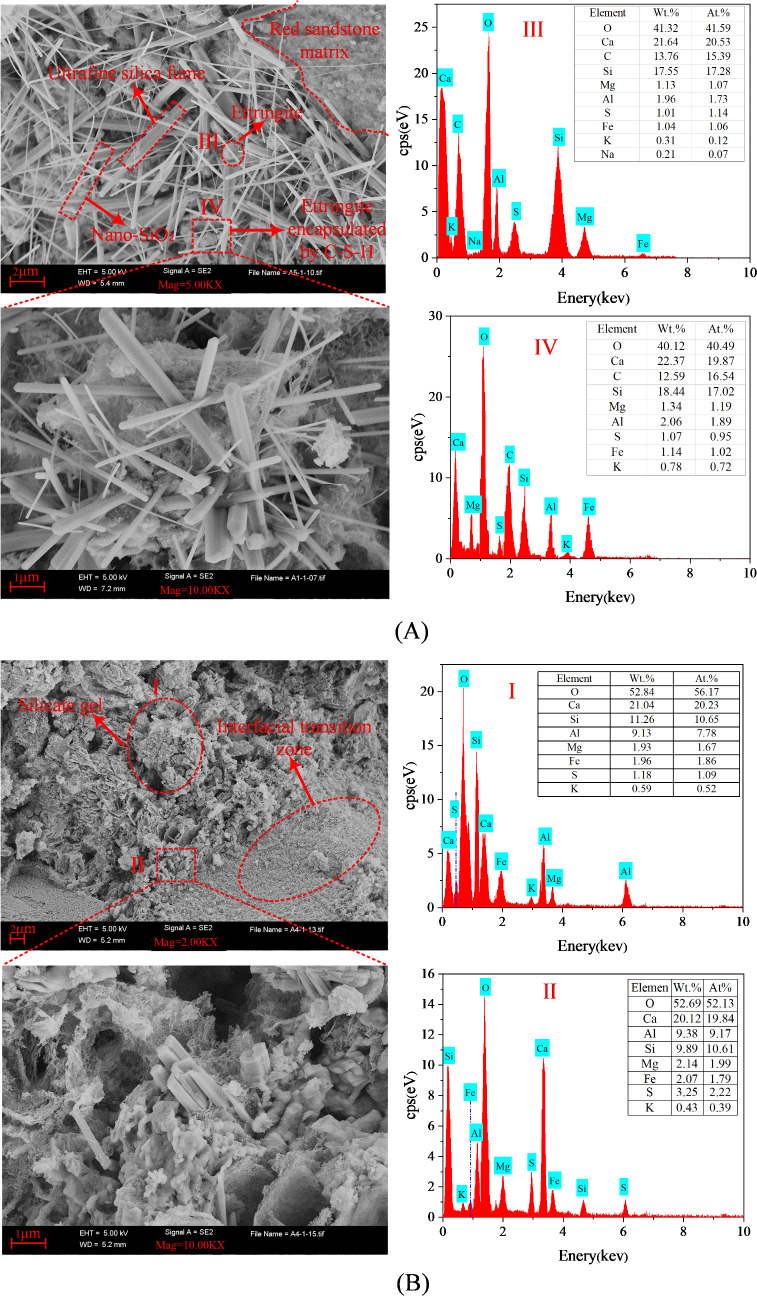
SEM images and EDS analysis of the grout-rock interface consolidated body in specimens. (A) SEM images and EDS analysis at points I and II of the grout-rock interface consolidated body in 4−2# disc specimen; (B) SEM images and EDS analysis at points III and IV of the grout-rock interface consolidated body in 2−2# disc specimen.

The micro-morphology and structural characteristics of the interface between the grout consolidated body and red sandstone can be clearly observed using scanning electron microscopy (SEM). Fig 12 shows the macro- and micro-morphology of fractured disc specimens reinforced with ordinary Portland cement grouting material and HFUES grouting material. As shown in Fig 12A, ordinary Portland cement grouting material generates a small amount of hydration products, such as C-S-H gel and ettringite, during the hydration process. These products mainly exhibit a loose flaky or gel-like structure (Fig 12A, Point I). According to EDS test results, the total content of Si and Ca elements in this region is approximately 32.30%. Additionally, a distinct interfacial transition zone is formed between the grout consolidated body and the sandstone matrix (Fig 12A, Point II). This zone consists of the grouting material, red sandstone mineral particles, and chemical reaction products between them, presenting a continuous gradient structure. EDS test results show that the total content of Si and Ca in the transition zone is approximately 30.01%, indicating relatively less C-S-H gel formation in this region, which aligns well with the macro-morphological characteristics of the grout-rock interface.

Compared to ordinary Portland cement grouting material, the HFUES grouting material exhibits significant improvements in fluidity, permeability, and reactivity, as shown in Fig 12B. By incorporating nano-SiO_2_ and modified ultrafine fly ash, the micro-pores in the cement-based grouting material are effectively filled, significantly reducing the porosity of the consolidated body and the fractured sandstone surface, while greatly enhancing the interfacial bonding strength between the grout consolidated body and the sandstone. Due to their high specific surface area and reactivity, nano-SiO_2_ and modified ultrafine fly ash significantly promote the hydration reaction of the grouting material, generating more hydration products, such as C-S-H gel. As shown in Fig 12B, Point III, a large number of ettringite particles are densely wrapped by a fibrous network of hydration products, with the Si and Ca content significantly increased to approximately 39.19%. Additionally, the Si and Ca content in the C-S-H gel reaches 40.81% (Fig 12B, Point IV). The formation of these hydration products significantly improves the density and continuity of the grout-rock interface, which is a key reason for the transformation of fractured red sandstone from a loose state to a dense and cohesive structure.

The strength development mechanism of silicate cement-based grouting materials is a complex multiscale process. Research indicates that the mechanical properties of hardened materials primarily depend on four key sub-microstructural characteristics: porosity and pore size distribution, solid-phase composition of hydration products, volume fraction of cementitious phases, and interfacial behavior. Based on SEM and CT analysis, the hardened slurry of high-fluidity ultra-early-strength grouting materials exhibits typical multiphase composite, porous medium, and multiscale hierarchical structural features. The microstructure demonstrates significant anisotropy and heterogeneity, primarily attributed to the synergistic effects of various additives and their differential contributions to strength enhancement. The mechanisms of individual components can be systematically elucidated as follows:

(1) Modified ultrafine fly ash influences material performance through a dual mechanism: On one hand, its high pozzolanic activity effectively accelerates cement hydration. On the other hand, ultrafine particles physically fill micron-scale pores. At an optimal dosage, the continuous formation of reaction products promotes a densified microstructure. However, excessive incorporation leads to Ca(OH)_2_ overaccumulation, thereby compromising strength.
(2) Ultrafine silica fume exhibits notable micro-nano scale effects: Physically, its nano-sized particles efficiently fill mesopores within C-S-H gels. Chemically, reactive components undergo secondary reactions with Ca(OH)_2_ in pore solution, generating additional C-S-H gels. These newly formed gels interlock with hexagonal prismatic calcium aluminate hydrate crystals, constructing a more stable three-dimensional network structure.
(3) Nano-SiO_2_ demonstrates unique time-dependent effects: In the early stage, its high specific surface area serves as nucleation sites for hydration products, forming a silicon-rich transition layer on cement particle surfaces. As hydration progresses, its latent pozzolanic activity gradually releases, consuming Ca(OH)_2_ to produce secondary calcium silicate hydrate gels, significantly reducing porosity and enhancing mechanical properties

From a material design perspective, these three components form a synergistic system with complementary advantages: Modified ultrafine fly ash provides sustained pozzolanic effects, ultrafine silica fume contributes immediate reactivity and nucleation promotion, while nano-SiO_2_ combines early nucleation with long-term reactivity. Coupled with their respective pore-filling effects, they optimize multiscale pore structure, regulate reaction products, and strengthen interfaces, ultimately achieving remarkable improvements in macroscopic mechanical performance. This multi-mechanism synergy provides a crucial theoretical basis for developing high-performance cement-based materials.

## Conclusions

(1) This study adopted a four-factor five-level orthogonal experimental design to systematically investigate the effects of four components—modified ultrafine fly ash, ultrafine silica fume, nano-SiO2, and polycarboxylate superplasticizer—on the performance of cement-based grouting materials. By testing 25 groups of mixtures for key properties including viscosity, fluidity, setting time, and hardened strength, combined with range sensitivity analysis and comprehensive balance analysis, the optimal formulation was determined as follows: 5.00 wt.% modified ultrafine fly ash, 10.00 wt.% ultrafine silica fume, 9.00 wt.% nano-SiO2, and 0.05 wt.% polycarboxylate superplasticizer. This formulation enables the preparation of ultrafine cement-based grouting materials with high fluidity and ultra-early strength characteristics.
(2) The high-flowability ultra-early-strength grouting material demonstrates significantly superior reinforcement effects on fractured sandstone compared to ordinary Portland cement grouting material. Specimens reinforced with the high-flowability material exhibit strain-hardening characteristics post-peak, whereas those treated with ordinary material display strain-softening behavior. Relative to the residual strength of intact specimens, the uniaxial compressive strength and tensile load capacity of high-flowability-reinforced specimens increased by 123.17% and 67.42%, respectively, reaching 2.38 times and 2.25 times those of ordinary grout-reinforced specimens. Furthermore, the elastic modulus of high-flowability-reinforced specimens recovered to 94.00% of intact specimens, markedly outperforming the 40.50% recovery observed with ordinary material. These results conclusively validate the outstanding advantages of high-flowability ultra-early-strength grouting material in enhancing the mechanical properties of fractured sandstone. The failure mode of high-flowability-reinforced specimens predominantly involves progressive tensile-shear failure, while ordinary material-reinforced specimens primarily exhibit brittle shear failure.
(3) CT analysis revealed that the stone body volume fraction in discs and standard specimens reinforced with high-flow ultra-early-strength grouting material was 1.33 times and 1.47 times higher, respectively, than those reinforced with ordinary Portland grouting material. Conversely, the fracture volume fraction was reduced to 0.36 times and 0.73 times, respectively. SEM and mineral composition tests demonstrated that the high-flow ultra-early-strength grouting material formed a fibrous network of hydration products at the grout-rock interface, with significantly increased Si and Ca element content. In contrast, ordinary Portland grouting material only produced loosely compacted flaky or gel-like products, accompanied by corresponding decreases in Si and Ca element content.
